# Superoxide Dismutase 1 Nanozyme for Treatment of Eye Inflammation

**DOI:** 10.1155/2016/5194239

**Published:** 2015-12-01

**Authors:** Olga A. Kost, Olga V. Beznos, Nina G. Davydova, Devika S. Manickam, Irina I. Nikolskaya, Anna E. Guller, Petr V. Binevski, Natalia B. Chesnokova, Anatoly B. Shekhter, Natalia L. Klyachko, Alexander V. Kabanov

**Affiliations:** ^1^Chemistry Faculty, M.V. Lomonosov Moscow State University, Moscow 119991, Russia; ^2^Helmholtz Institute for Eye Disease, Moscow 105062, Russia; ^3^UNC Eshelman School of Pharmacy, University of North Carolina at Chapel Hill, Chapel Hill, NC 27599-7362, USA; ^4^Macquarie University, Sydney, NSW 2109, Australia; ^5^Research Institute for Molecular Medicine, I.M. Sechenov First Moscow State Medical University, Moscow 119992, Russia; ^6^Pirogov Russian National Research Medical University, Moscow 117997, Russia

## Abstract

Use of antioxidants to mitigate oxidative stress during ocular inflammatory diseases has shown therapeutic potential. This work examines a nanoscale therapeutic modality for the eye on the base of antioxidant enzyme, superoxide dismutase 1 (SOD1), termed “nanozyme.” The nanozyme is produced by electrostatic coupling of the SOD1 with a cationic block copolymer, poly(L-lysine)-poly(ethyleneglycol), followed by covalent cross-linking of the complexes with 3,3′-dithiobis(sulfosuccinimidylpropionate) sodium salt. The ability of SOD1 nanozyme as well as the native SOD1 to reduce inflammatory processes in the eye was examined *in vivo* in rabbits with immunogenic uveitis. Results suggested that topical instillations of both enzyme forms demonstrated anti-inflammatory activity; however, the nanozyme was much more effective compared to the free enzyme in decreasing uveitis manifestations. In particular, we noted statistically significant differences in such inflammatory signs in the eye as the intensities of corneal and iris edema, hyperemia of conjunctiva, lens opacity, fibrin clots, and the protein content in aqueous humor. Clinical findings were confirmed by histological data. Thus, SOD1-containing nanozyme is potentially useful therapeutic agent for the treatment of ocular inflammatory disorders.

## 1. Introduction

Uveitis is an inflammatory disease of the uvea, a section of the eye which consists of the middle pigmented vascular structures of the eye and includes the iris, ciliary body, and choroid. Common causes of uveitis include infections, multisystem disorders such as sarcoidosis and Behçet's disease and autoimmune disorders such as rheumatoid arthritis or ankylosing spondylitis [1–4]. Uveitis is a severe sight threatening disease, frequently leading to vision loss and blindness with retinal vasculitis, retinal detachment, and glaucoma. Uveitis accounts for 5–20% of legal blindness in United States and in Europe, and perhaps as much as 25% of blindness in the developing world [1]. Severe cases of uveitis need to be treated aggressively to prevent damage caused by chronic inflammation. Corticosteroids constitute the first line of therapy for patients with noninfectious ocular inflammatory disease. However, as the use of corticosteroids became more prevalent in treating ocular inflammation, the side effects of this treatment became more prevalent as well. Another class of compounds, known as “immunosuppressive drugs,” such as cyclosporine A, was found to be successful in treating uveitis. However, such treatment is also complicated by side effects associated with immunosuppression [5–7]. Patients who cannot take medications because of the side effects or patients who are not responsive to the existing medications experience unavoidable impaired visual function. Thus, it is important to investigate alternative approaches for the treatment of uveitis.

Inflammatory diseases, including ocular ones, are accompanied by excessive production of reactive oxygen species (ROS) and by depletion of endogenous antioxidants. Antioxidant enzymes, superoxide dismutase 1 (SOD1, also known as Cu/Zn SOD), catalase, and glutathione peroxidase are known to be very effective scavengers of ROS. These enzymes were shown to be effective in the treatment of various eye diseases associated with oxidative stress. Thus, SOD1 was used for the treatment of lens-induced and bovine albumin-induced uveitis in rabbits [8, 9], as well as for the treatment of acute corneal inflammation in animals induced by sodium hydroxide [10, 11]. Both SOD1 and glutathione peroxidase were employed for the treatment of severe experimental allergic uveitis induced by retinal S antigen in rats [12], while poly(ethylene glycol)- (PEG-) modified catalase and PEG-SOD were employed for the treatment of the same type of uveitis in guinea pigs [13]. We have shown recently [14] that SOD1 instillations may help to reduce clinical presentations of immunogenic uveitis in rabbits.

Eye diseases are most commonly and preferably treated by topical instillations of eye drops. These formulations face technical and clinical problems, such as solubility of the components and instability of drug solutions, limited efficacy and limited corneal/sclera permeability, and local and systemic toxicity. Moreover, 2 min after instillation the major part of the topical drug solution is eliminated via the nasolacrimal drainage system limiting ocular penetration of the drug to less than 5% of the administered dose [15].

Nanoparticles are colloidal drug carrier systems that can improve the efficacy of drug delivery into the eye by overcoming corneal/sclera diffusion barrier. Drug loaded polymeric nanoparticles offer several favorable biological properties, such as biocompatibility and mucoadhesiveness, enhancing bioavailability without blurring the vision. The use of drug-containing nanoparticles can decrease the dose of the drug and diminish side effects. So, nanoparticles are a promising drug delivery system, which fulfills the requirements for ophthalmic application (for reviews, see [16, 17]).

Recently, new formulations of antioxidant enzymes, SOD1 and catalase, were prepared by electrostatic coupling of these negatively charged enzymes (pI values are 4.95 and 5.8 for SOD1 and catalase, resp.) with cationic block copolymers, such as methoxy-PEG-*block*-poly(L-lysine hydrochloride) block copolymer (PEG-pLL_50_), followed by covalent cross-linking to stabilize nanoparticles. Catalytic nanoparticles based on polyion complexes of enzymes with block copolymers of opposite charge were termed “nanozymes” [18–22]. Spontaneous self-assembly of oppositely charged proteins and polymers results in stoichiometric complexes with 100% loading efficiency. These nanozymes were shown to be prospective agents for the treatment of various diseases of the central nervous system due to prolonged ability to scavenge experimentally induced ROS in cultured brain microvessel endothelial cells and central neurons, increased stability in both blood and brain, enhanced penetration through the blood-brain barrier, and, therefore, increased accumulation in brain tissues, in comparison with non-cross-linked complexes and native enzyme [18, 21, 22].

In the current study, we demonstrate the advantages of topical instillations of superoxide dismutase 1 in the form of “nanozyme” in the treatment of ocular inflammation in a rabbit model of immunogenic uveitis.

## 2. Methods

### 2.1. Preparation of and Characteristics of Nanozyme

SOD1 nanozyme was synthesized by self-assembly of recombinant SOD1 (“Enzyme Technologies”, St. Petersburg, Russia) with cationic block copolymer, PEG-pLL_50_ (MW 13 kDa, polydispersity index 1.09, Alamanda Polymers, Huntsville, AL) in aqueous solution followed by cross-linking with 3,3′-dithiobis(sulfosuccinimidylpropionate) (DTSSP) as in [21]. Unreacted cross-linker was desalted using NAP-25 column, and cross-linked nanozymes were purified using a 100 kDa MWCO filter. Purified particles were then lyophilized from 0.05 M Hepes-buffer, pH 7.5, containing 0.15 M NaCl, and stored at −20°C. For further experiments, precalculated quantity of lyophilized nanozyme was dissolved in deionized water and gently vortexed for 2 min until sample dissolved completely. Intensity-mean z-averaged particle diameter (effective diameter), polydispersity index (PDI), and *ζ*-potential were measured after filtration via a 0.2 *μ*m filter using a Zetasizer Nano ZS (Malvern Instruments Ltd., MA). Aliquots of nanozyme solution required for daily experiments were then frozen and kept at −20°C.

### 2.2. Enzyme Activity

SOD1 activity was determined using SOD1 ability to inhibit autooxidation of quercetin as in [23] with detection kit (Belarusian State University, Belarus). The experimental sample in phosphate buffer, pH 7.8, containing 0.08 M EDTA, 0.125% (v/v) TEMED, was mixed with quercetin solution in DMSO. The absorbance was measured at 406 nm immediately after addition of quercetin (*D*_0_) and after 20 min. (*D*_20_). In control, phosphate buffer was used instead of the sample, and the absorbances ^*c*^ *D*_0_ and ^*c*^ *D*_20_ were measured, correspondingly. The percent of inhibition of quercetin autoxidation by SOD1 in experimental samples was calculated by the formula [(^*c*^ *D*_0_ − ^*c*^ *D*_20_)−(*D*_0_ − *D*_20_)]/(^*c*^ *D*_0_ − ^*c*^ *D*_20_) × 100. One unit of SOD1 activity was defined as the amount of SOD1, which inhibits the quercetin autoxidation by 50%. The protein content was determined using Micro BCA Protein Assay Kit (Pierce, Rockford, IL).

### 2.3. *In Vitro* Drug Release Study

One mg of SOD1 nanozyme was dispersed in 0.5 mL of PBS, transferred to 100 kDa MWCO membrane, and centrifuged for 5 min at 1200 ×g. Supernatant (about 0.05 mL) was diluted by PBS to the initial volume and centrifuged again. The “filtration-dilution” procedure was repeated 5 times. SOD1 activity and protein concentration were measured in the initial nanozyme solution, in each filtrate, and in the final supernatant. In another series of experiments, equal amounts of freshly dissolved nanozyme in PBS were incubated at room temperature for different time periods. Then, the solutions were filtered through 100 kDa membrane, and SOD1 activity in the filtrates was determined.

### 2.4. Animals

A randomized and double-blinded study was conducted using adult Chinchilla rabbits weighing 2.0–2.5 kg. All experiments with live rabbits were carried out in strict accordance with the Association for Research in Vision and Ophthalmology (ARVO) statement for the Use of Animals in Ophthalmic and Vision Research. The protocol was approved by the Committee on the Ethics of Animal Experiments of the Helmholtz Institute for Eye Disease (Permit number 22/2). All efforts were made to minimize rabbits suffering. After the end of experiments the rabbits were sacrificed by lethal pentobarbital injection.

### 2.5. *In Vivo* Studies

Immunogenic uveitis was induced as described in [24]. Briefly, rabbits were initially injected subcutaneously with 5 mL of normal horse serum for sensitization. Ten days later, 5% anesthetic Alcain (Alcon, Belgium) was instilled into each eye before the intravitreal injections of 70 *μ*L of the same serum in the eyes to induce acute uveitis.

Rabbits received 30 *μ*L of the drug solutions as eye drops topically in each eye three times a day for 14 days. Three independent series of experiments for clinical estimation of uveitis were performed. In each series, animals were randomly divided into 4 groups (*n* = 5 per each group, i.e., 10 eyes) and treated as follows: (1)* control* (healthy) group without uveitis and (2)* placebo* group with uveitis received 10 mM Hepes buffer, pH 7.4, containing 0.15 M NaCl; (3)* SOD1* group with uveitis received 1 mg/mL SOD1 solution in the same buffer; (4)* treatment (nanozyme)* group with uveitis received 8–10 mg/mL SOD1 nanozyme solution in the same buffer. Hepes buffer did not cause any irritation of the eye. SOD1 dose was chosen in accordance with what was recommended in [25]. The dose of nanozyme solution was calculated based on enzymatic activity of nanozyme (units per mg) so that the activities of SOD1 and nanozyme solutions were equal; that is, 8–10 mg nanozyme corresponded to 1 mg SOD1 by specific activity. Eyes were examined in a double-blinded trial by indirect ophthalmoscopy using a slit lamp (Zeiss slit lamp 30SL, USA). Clinical symptoms of uveitis, including eyelid and conjunctival edema and hyperemia, corneal edema and neovascularization, iris edema and hyperemia, fibrin clots and precipitates on the iris and on the lens, lens opacity, presence of synechiae (cohesions between the pupillary margin of iris and anterior part of the lens), which lead to immobilization of the pupil, and presence of purulent exudate (hypopyon) and blood (hyphema) in the anterior chamber of the eye, were estimated. Evaluation of inflammation scores was performed using a conventional scale: (0) no symptom; (1) low degree of manifestation; (2) medium; (3) strong.

### 2.6. Analyses of Aqueous Humor

Aqueous humor (intraocular fluid) from anterior chamber of the eye was collected by paracentesis in limb area under topical anesthesia on day 8 of uveitis (that is, 16 h after last instillations of the drugs) and on day 4 in the separate experiment on two rabbits (4 eyes) in each group. The samples were centrifuged at 21,000 ×g for 10 min, and the supernatant was stored at −20°C. The amount of leukocytes was determined microscopically. The *α*_2_-macroglobulin content (in arbitrary units) was estimated indirectly as previously described [26, 27] based on the ability of the complex of *α*_2_-macroglobulin with trypsin to react with benzoyl-L-arginine-p-nitroanilide as a substrate. Antioxidant activity was determined by chemiluminescence kinetics in hemoglobin-H_2_O_2_-luminol system as described in [28] with Trolox, a water-soluble analog of vitamin E, as a standard antioxidant. Antioxidant activity of the sample was expressed as trolox-equivalents calculated on the basis of a trolox standard curve.

### 2.7. Histopathology

Histopathological analysis was performed in a double-blinded fashion. For this experiment, we used 10 rabbits (20 eyes), 9 rabbits with uveitis and 1 healthy rabbit. For the topical treatment, rabbits with uveitis were randomly divided into three groups. The 1st group of 3 rabbits received placebo as described above, the 2nd group of 3 rabbits received native SOD1, and the 3rd group of 3 rabbits received SOD1 nanozyme with the same SOD1 activity. On day 4 rabbits were sacrificed by sodium pentobarbital injection (100 mg/kg), and the eyes were enucleated. Samples were fixed in 10% neutral buffered formalin, dehydrated in a graded series of alcohol, embedded in paraffin, and cut into 4-5 *μ*m serial sections. The sections were stained with haematoxylin and eosin (H&E) and examined in upright light microscope (Olympus BX51) using dry-air (4./NA0.10; 10./NA0.25; 20./NA0.40) and oil-immersion (100./NA1.25 oil) objectives (Olympus Optical, Tokyo, Japan). Histology images were recorded in a single-frame mode using a digital video camera SDU-252 (2048 × 1536, “Spetsteletechnika”, Russia) integrated into the microscope optical path.

### 2.8. Statistical Analysis

All data are means ± SEM. Significance was analyzed using the Mann-Whitney* U* test with STATISTICA 6 (StatSoft, Inc., OK).

## 3. Results

### 3.1. Synthesis and Characterization of Nanozyme

SOD1 nanozyme was synthesized as described earlier [21] by mixing of aqueous solutions of SOD1 and block copolymer, PEG-pLL_50_ at pH 7.4 followed by cross-linking and purification. SOD1 retained 100% its catalytic activity in polyion complex before cross-linking consistent with previous report [21, 22] but partly lost activity as a result of cross-linking with DTSSP and filtering through 100 kDa membrane. Altogether, the lyophilized dry nanozyme samples displayed the specific activity about 30 kU/mg, while the activity of the unmodified pure recombinant SOD1 was ca. 250 kU/mg. The observed decrease in the specific activity was mainly due to the presence of the bulk of polymer in nanozyme as well as to the presence of buffer substance and salt in the final lyophilized preparation. The DLS analysis revealed that the particles of SOD1 nanozyme had an effective diameter of 35 nm (compared to about 5 nm for native SOD1, as reported in [21]), narrow particle size distribution (PDI ca. 0.1), and nearly neutral (zero) *ζ*-potential.

**Figure 1:**
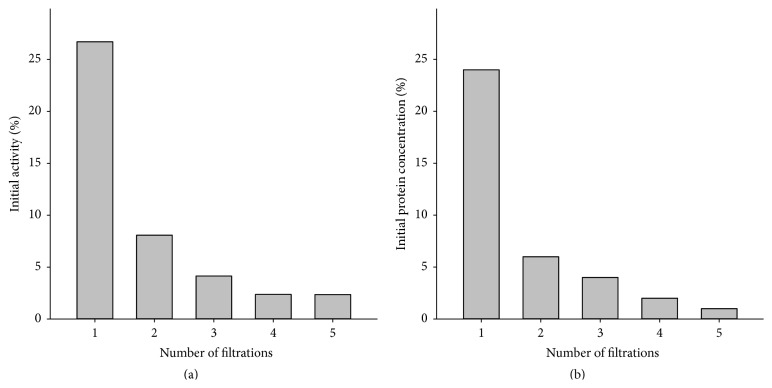
The release of SOD1 activity and protein upon subsequent dilutions and filtrations of nanozyme. Nanozyme was dissolved in deionized water and immediately filtered through 100 kDa MWCO filter, supernatant was diluted using PBS to the initial volume and filtered again. “Dilution-filtration” steps were repeated, and SOD activity (a) and protein content (b) were determined in each filtrate. The experiment was performed in duplicate.

To examine whether SOD1 can be released from the nanozyme we determined the activity of SOD1 in the filtrates and supernatant (1) after repeated centrifugal filtration of the nanozyme solution using 100 kDa MWCO filters and (2) after centrifugal filtration of nanozyme aqueous solution incubated for different periods of time. In the first experiment, the lyophilized nanozyme was dissolved in deionized water so that the final concentration of NaCl was 0.15 M and immediately filtered through 100 kDa membrane for 5 min. After this first filtration step, about 24% of the protein and about 27% of SOD1 activity (Figure 1) were found in the filtrate. Further dilutions of supernatant to the initial volume and subsequent filtration resulted in additional release of SOD1 activity and protein from the nanozyme, albeit to a lesser extent than the initial filtration step, from 2 to 7% of the initial amount. After 5 subsequent dilutions and filtrations, the nanozyme retained about 55–65% of both SOD1 activity and protein. It is interesting that the release of SOD1 from the nanozyme occurs not only after the “dilution-filtration-dilution” procedures but also upon incubation of its aqueous solution for various time periods after preparation. Specifically, freshly prepared solution of 2 mg nanozyme in 0.5 mL 0.15 M NaCl contained about 25 to 30% free SOD1, while after 2 hr incubation the same solution contained 40% of the free SOD1, and 4 hr 40 to 50% of the free SOD1. Further incubation of the nanozyme solution did not result in the further release of the free SOD1. These data suggest that the nanozyme synthesis process and specific chemistries used in this work produce nanozymes encapsulating significant portion of SOD1 that is not chemically coupled to the block copolymer and can be released in the surrounding media as it was observed previously [22].

### 3.2. Effects of Topical Instillations of SOD1 and SOD1 Nanozyme on Clinical Manifestations of Immunogenic Uveitis in Rabbits

We induced immunogenic uveitis in rabbits in three independent series of experiments. In one series, the uveitis appeared to show severe manifestations of inflammation in the outer part of the eye (eyelid, cornea, and conjunctiva), while, in the other two, inflammation of these tissues was rather moderate. Manifestations of inflammation in the inner part of the anterior segment of the eye, however, were significant in all three series of experiments. It is noteworthy that, in all series, uveitis developed similarly, with the most acute phase on days 3–5 and fading till the end of second week.

#### 3.2.1. Rabbits without Treatment (Placebo)

Three days past intravitreal injection of horse serum, the eyes of animals showed classical clinical symptoms of anterior uveitis which intensified on day 4. Edema of the eyelid, cornea, and conjunctiva were observed. Hyperemia of conjunctiva was significant. Iris had both edema and hyperemia; its structure was changed. There was a lot of fibrin clots in the anterior part of the eye, which, in several cases, formed massive clouds. In most eyes, there were multiple synechiae, which resulted in pupil immobilization, improper pupil form, and the lack of reaction of pupil to light. Fibrin clots were also found on the surface of lens of all eyes; lenses itself were characterized by significant opacity, which thwarted microscopic investigation of the vitreous body. Many animals (about half) had massive purulent exudates (so-called hypopyon) in the anterior camera of the eye, which was formed by leukocytes and detritus. Neovascularization of the cornea, which is known to be a result of oxidative stress [29], was observed in half of the eyes. Some animals exhibited symptoms of elevated intraocular blood pressure (from 8th day) which indicated the development of common uveitis complication, secondary glaucoma.

#### 3.2.2. SOD1-Treated Rabbits

**Figure 2:**
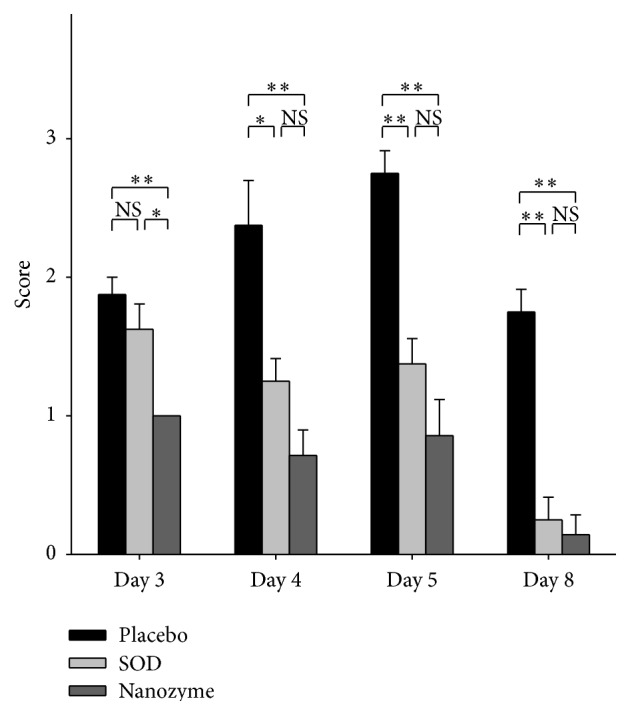
The effect of instillations of nanozyme and SOD solutions in the rabbit eye on conjunctival hyperemia at uveitis. The data from three series of experiments were analyzed, each experiment including 5 animals (10 eyes) in each group: control, placebo, SOD1-treated, and SOD1 nanozyme-treated group. Thus, *n* = 30 for each group. The scores were estimated as a degree of manifestation of hyperemia of conjunctiva: 0: no symptom; 1: low degree of manifestation; 2: medium degree; 3: strong degree. Symbols: *∗*: the level of significance of differences by the Mann-Whitney *U* test *p* < 0.05; *∗∗*: the level of significance of differences by the Mann-Whitney *U* test *p* < 0.01; NS: not significant differences.

**Figure 3:**
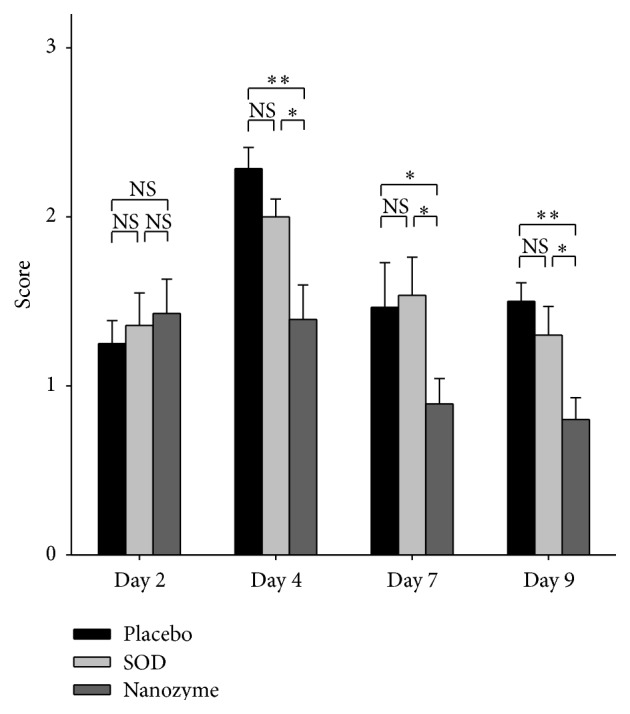
The effect of instillations of nanozyme and SOD solutions in the rabbit eye on the formation of fibrin clots at uveitis. The data from three series of experiments were analyzed, each experiment including 5 animals (10 eyes) in each group: control, placebo, SOD1-treated, and SOD1 nanozyme-treated group. Thus, *n* = 30 for each group. The scores were estimated as degree of fibrin clots formation: 0: no clots; 1: low degree of clot formation; 2: medium degree; 3: strong degree. Symbols: *∗*: the level of significance of differences by the Mann-Whitney *U* test *p* < 0.05; *∗∗*: the level of significance of differences by the Mann-Whitney *U* test *p* < 0.01; NS: not significant differences.

The development of uveitis in this group remarkably differed from that in placebo group. Eyelid edema was much less pronounced, and hypopyon was absent at all times during the disease. Corneal and iris edema were only local and diminished in time. Conjunctival edema and hyperemia were less pronounced as well. Neovascularization of the cornea in the acute phase of uveitis was observed in 20–30% of eyes. During treatment, we observed regress of synechiae formation (from day 4 to day 8) and partial restoration of the reaction of the pupil to light. At the end of the treatment, however, lens opacity decreased insignificantly. Many eyes retained precipitates on the lens. Figures 2 and 3 are representative examples of the comparative effects of SOD1 and placebo instillations in the rabbit eye on conjunctival hyperemia and formation of fibrin clots at different times during uveitis. It was seen that while there was no statistical difference between the extents of hyperemia of conjunctiva in the eyes of SOD-treated and placebo-treated eyes on day 3, later (on days 4 and 5) this difference became statistically significant and on day 8 this difference is remarkable (Figure 2). On the contrary, there was no difference between the amount of fibrin clots observed in SOD-treated and placebo-treated rabbits (Figure 3).

#### 3.2.3. SOD1 Nanozyme-Treated Rabbits

Clinical manifestations of uveitis in this group were less pronounced and appeared later than that in placebo- and SOD1-treated groups. Most importantly, hyperemia of conjunctiva, corneal edema, iris edema, and lens opacity were significantly less pronounced than in the two other groups. There were no eyes with neovascularization of the cornea in this group. Synechiae were lower by 20–25%, which improved the pupil reaction to light. Fibrin clots were less intense as well. Figure 2 demonstrates the effect of SOD1 nanozyme instillations on the conjunctival hyperemia in comparison with the effects of instillations of placebo and native SOD1. There was a clear statistical difference (*p* < 0.01) between SOD1 nanozyme and placebo groups at all times of the disease. Moreover, nanozyme was statistically more effective than native SOD1 on day 3 (in acute phase of uveitis). The formation of fibrin clots in the case of nanozyme-treated groups was statistically less pronounced (Figure 3) than in the placebo- and SOD1-treated groups at every time point of uveitis, starting from day 4. Thus, we demonstrated that nanozyme treatment resulted in the considerable improvement of uveitis condition in rabbits compared not only with the untreated animals but with the native SOD1-treated group as well.

### 3.3. Effects of Topical Instillations of SOD1 and SOD1 Nanozyme on Clinical Symptoms of Uveitis in the Acute Phase of the Disease

**Figure 4:**
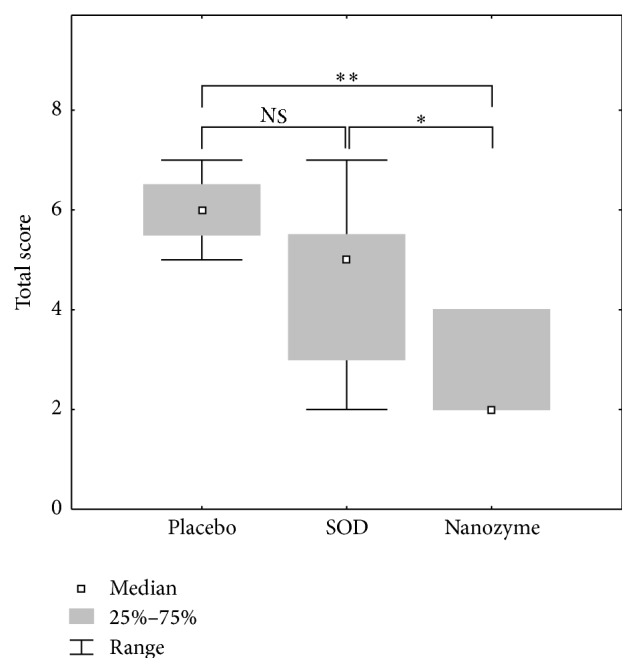
Comparison of the clinical symptoms of uveitis in external rabbit eye structures as a sum of the scores for eyelid edema, conjunctival hyperemia, and corneal edema in the acute phase of the uveitis. The data from three series of experiments were analyzed, each experiment including 5 animals (10 eyes) in each group: control, placebo, SOD1-treated, and SOD1 nanozyme-treated group. Thus, *n* = 30 for each group. The scores were estimated as degrees of manifestations of clinical symptoms of the disease: 0: no symptom; 1: low degree of manifestation; 2: medium degree; 3: strong degree. Symbols: *∗*: the level of significance of differences by the Mann-Whitney *U* test *p* < 0.05; *∗∗*: the level of significance of differences by the Mann-Whitney *U* test *p* < 0.01; NS: not significant differences.

**Figure 5:**
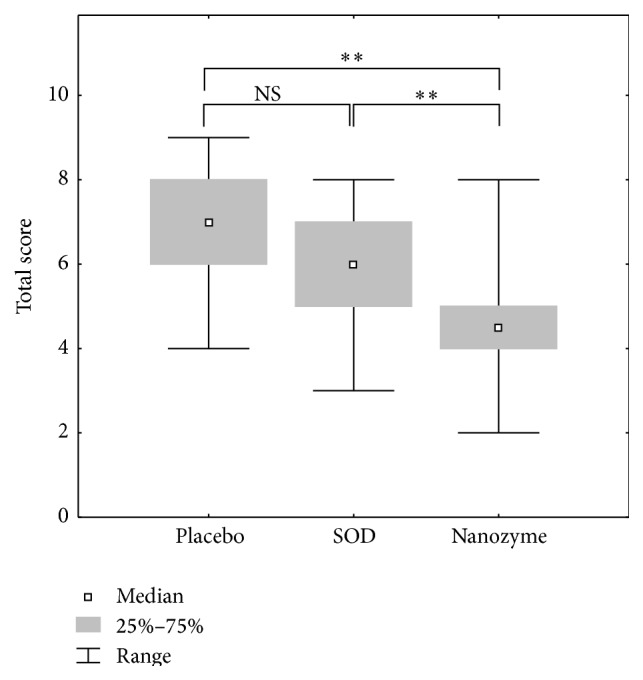
Comparison of the clinical symptoms of uveitis in internal rabbit eye structures as a sum of the scores for iris edema, fibrin clots, and lens opacity in the acute phase of the uveitis. The data from three series of experiments were analyzed, each experiment including 5 animals (10 eyes) in each group: control, placebo, SOD1-treated, and SOD1 nanozyme-treated group. Thus, *n* = 30 for each group. The scores were estimated as in the legend of Figure 3. Symbols: *∗∗*: the level of significance of differences by the Mann-Whitney* U* test *p* < 0.01; NS: not significant differences.

Most clearly, the differences in the effects of topical instillations of SOD1 nanozyme, SOD1, and placebo are seen in the acute phase of the disease, that is, on days 3-4. We compared the efficacy of these treatments using the sums of the scores for the manifestations of inflammation in the outer and inner parts of the anterior eye segment. This is a common approach in ophthalmology to test drug efficacy [30]. The manifestations of inflammation were assessed by (1) the eyelid edema, corneal edema, and hyperemia of conjunctiva in the outer part and (2) the iris edema, lens opacity, and fibrin clots in the inner part. The results are shown in Figures 4 and 5. While native SOD1 seems to have a healing effect on inflammation in the outer part of the eye, this effect was not statistically significant. In contrast, SOD1 nanozyme showed statistically significant healing effect (Figure 4) in comparison with both placebo (*p* < 0.01) and native SOD1 (*p* < 0.05). The difference between SOD1 formats was even more pronounced when we compared their effect on the inflammation in the inner part of the anterior segment of the eye (Figure 5). While the healing effect of native SOD1 was not statistically different from that of placebo, the SOD1 nanozyme showed remarkable healing effect, which was significantly distinct from the effects of native SOD1 and placebo (*p* < 0.01 in both cases).

### 3.4. Effect of Treatments on the Leukocyte Counts and Biochemical Parameter of the Aqueous Humor of the Eye

**Figure 6:**
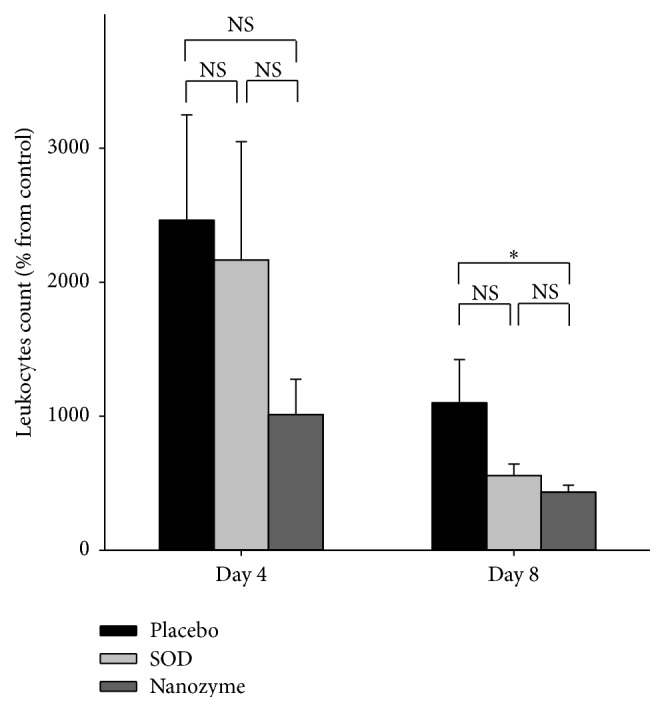
Leukocyte numbers in aqueous humor of the eye of uveitis rabbits as percentage from the value for control (healthy) rabbits on different times of the disease. *n* = 4 in each group on day 8; *n* = 10 in each group on day 8.

ROS metabolites are predominantly produced by polymorphonuclear leukocytes, which migrate to inflamed tissues and can serve as an indication of the inflammation. The aqueous humors from the eyes of all rabbits with uveitis contained considerable amount of leukocytes both in the acute (day 4) and later (day 8, e.g., after 16 h after the last instillations of the drugs) phases of the disease. On day 4, the SOD1-treated group displayed approximately the same leukocyte counts as placebo group, while SOD1 nanozyme-treated group exhibited decrease in leukocyte counts although statistically insignificant. On day 8, both SOD-treated groups showed the decrease in leukocyte counts, SOD1 nanozyme-treated group exhibiting statistically significant effect compared with placebo (Figure 6).

Tissue inflammation is characterized by elevated total protein concentration in biological fluids along with the increase in proteinase inhibitor *α*_2_-macroglobulin and decrease of overall antioxidant activity, as well as increase in endogenous SOD1 [31–34]. Therefore, we determined these biochemical parameters in aqueous humor on day 8 after the disease onset (Table 1). The protein concentration in nontreated group increased 8-fold compared to healthy animals and it was only slightly affected after the native SOD1 treatment. In contrast, SOD1 nanozyme treatment resulted in nearly 2-fold decrease in this parameter. The effect of nanozyme was significant compared to both placebo and native SOD1 (*p* < 0.01). The development of uveitis also resulted in drastic increase of *α*_2_-macroglobulin activity in aqueous humor; however it was mitigated after both the native SOD1 and, especially, SOD1 nanozyme treatments (Table 1). Antioxidant activity increase in both SOD1 and nanozyme-treated groups was rather small and not significant (Table 1). The endogeneous SOD1 activity in aqueous humor of placebo-treated eyes with uveitis was increased by more than in 1.5 times compared to control group suggesting a compensatory reaction of the eye to the uveitis-induced oxidative stress. The native SOD1 treatments decreased the enzyme activity in aqueous humor, albeit nonsignificant, while SOD1 nanozyme treatment decreased this parameter significantly (Table 1). Notably, in the healthy and placebo-treated rabbits, SOD1 activity in aqueous humor represents only the endogenous enzyme, while in SOD1- and nanozyme-treated rabbits it may contain contributions of exogenous SOD1 as well. Still the decrease in the measured SOD1 activity in treated eyes clearly shows that SOD1 and, especially, nanozyme treatments decrease inflammation during experimental uveitis.

### 3.5. Histology Examination of the Disease Manifestation

**Figure 7:**
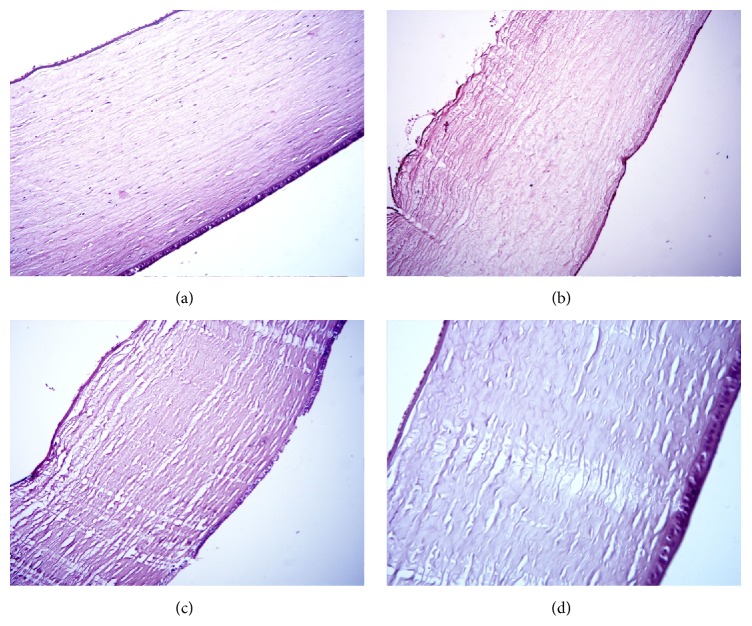
Histology picture of the cornea, stained with hematoxylin and eosin, magnification ×200, on day 4 of uveitis. (a) Control (*n* = 2): cornea is lined with epithelium and endothelium; collagen fibers and keratocytes (specialized corneal fibroblasts) are visible; (b) placebo (*n* = 6): swelling and loosening of corneal collagen fibers, endothelial desquamation, partial destruction of Descemet's membrane; epithelium is not changed; (c) SOD1 (*n* = 6): moderate loosening of the corneal collagen fibers, partial endothelial desquamation, cell infiltration is absent; (d) SOD1 nanozyme (*n* = 6): normal structure of the cornea.

**Figure 8:**
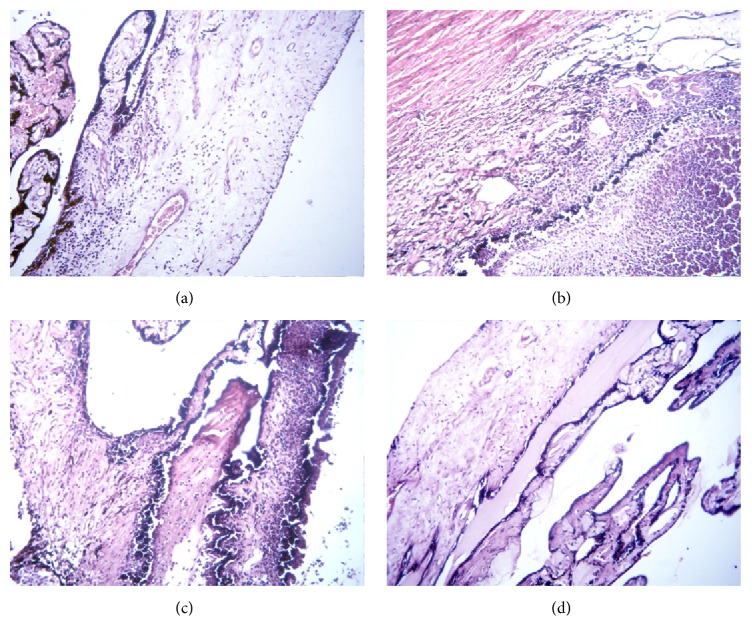
Histological picture of the ciliary body, stained with hematoxylin and eosin, magnification ×200, on day 4 of uveitis. (a) Control (*n* = 2): ciliary body with processes, moderate plethora; (b) placebo (*n* = 6): edema and infiltration of the ciliary body, deposition of purulent exudates; (c) SOD1 (*n* = 6): edema and inflammatory infiltration of the ciliary body; (d) SOD1 nanozyme (*n* = 6): ciliary body without cellular infiltration.

The eyes of the control, healthy rabbits were unchanged. The cornea displayed its common structure with thin multilayer epithelium on the outer side (cells form 2 or 3 layers) and single-layer endothelium on the inner side (Figure 7(a)). The epithelium and endothelium cells had normal structure; the main part of the stroma could be clearly seen. The conjunctival tissue was loose, moderately full-blooded. The vessels in the region of conjunction of cornea, conjunctiva, and sclera were wide and full-blooded as well. The sclera and ciliary body (Figure 8(a)) possessed their normal structure as well. The retina in the eyes of normal rabbits was also unchanged.

#### 3.5.1. Rabbits without Treatment (Placebo)

In the placebo group having uveitis, one eye contained white, thick, nontransparent expanding mass. This mass is known to represent a purulent exudate, consisting of leukocytes (some of them in a stage of disintegration) and small amount of fibrin. The vitreous body in this eye was in the state of destruction and cell infiltration. Another eye of the same animal, as well as the eyes of other rabbits in this group, maintained the vitreous body but the inner part of choroid contained white precipitates. Choroid in these eyes was thickened, known to be due to infiltration of neutrophils, macrophages, and lymphocytes. Some destruction of the pigment cells layer was also observed. The vessels within choroid were full-blooded but with some extent of erythrocyte aggregation. The cornea in this group exhibited edema, swelling, and loosening of collagen fibers, as well as partial desquamation of endothelium and partial destruction of Descemet membrane (Figure 7(b)). Epithelium of the cornea was not changed. The sclera in almost all eyes exhibited pronounced edema and contained sporadic neutrophil-macrophage infiltrates. The bundles of collagen fibers within sclera were loosened. There were also significant edema and inflammatory infiltration in ciliary body (Figure 8(b)), while loosened stroma of ciliary body exhibited signs of cellular dystrophy, characterized by the formation of cytoplasm vacuoles. Partial destruction and desquamation of epithelium of ciliary body and its processes with deposits of purulent exudates was also observed. Retina in the eyes of rabbits from this group had regions of destruction and exhibited signs of dystrophy of cells elements.

#### 3.5.2. SOD1-Treated Rabbits

The eyes of rabbits in the SOD1 treatment group had only moderate inflammation manifestation in uveal tract. The cornea was lined by unchanged epithelium. The Descemet membrane did not have defects; however, some regions of the cornea contained loosened collagen fibrils, and the endothelium was partially desquamated (Figure 7(c)). The sclera exhibited moderate edema and looseness with some inflammatory infiltration. The choroid was relatively thin, without purulent exudates but with slight infiltration by neutrophils, lymphocytes, and macrophages. Some eyes, however, contained thickened regions of choroid with more pronounced infiltrates, especially in the posterior segment of the eye. The ciliary body retained its ordinary structure, but exhibited some regions of edema and inflammatory cell infiltration (Figure 8(c)).

#### 3.5.3. SOD1 Nanozyme-Treated Rabbits

In the SOD1 nanozyme-treated group, 2 eyes (from 6 eyes examined) hardly showed any inflammatory symptoms. These eyes appeared to be unaffected by the disease and were indistinguishable of the eyes of the healthy rabbits. In the remaining eyes the cornea was laid by epithelium and endothelium without desquamation; the collagen fibers and keratocytes of stroma showed no changes as well (Figure 7(d)). The sclera in the eyes of this group was of common thickness without inflammation and loosening. The choroid was thin without destruction and cell infiltration. The retina was also unchanged. The ciliary body also displayed its natural structure, without signs of infiltration (Figure 8(d)).

Thus, while native SOD1 showed a pronounced therapeutic effect in the treatment of experimental immunogenic uveitis in rabbits, the nanoformulated form, SOD1 nanozyme, provided much more remarkable effect as revealed by the histopathology analysis.

## 4. Discussion

ROS are excessively produced in many disease states and contribute to tissue degeneration and pathogenesis of many clinical conditions including atherosclerosis, stroke, ischemia/reperfusion injury, myocardial infarction, central nervous system disorders, and wounds. In particular, ROS metabolites may be important factors in the early tissue damage that develops from immunopathologic inflammations [35–37]. Uncontrolled ROS production in acute inflammation can lead to destruction of structural and functional proteins as well as lipids in cell membranes. Because of the nonspecific nature of ROS-induced tissue injury, excessive release of these agents can cause substantial damage not only to the tissue in an inflamed state but also to the surrounding normal tissue. In particular, this is very important for the eye, as the transparency of the cornea and lens, as well as the functioning of photoreceptor apparatus, relies on their highly ordered structures, and excessive tissue damage will compromise visual function.

Antioxidants, SOD1 in particular, are known to be beneficial in the treatment of the various diseases connected with oxidative stress. Thus, SOD1 was reported to reduce inflammation [38], accelerate the healing of skin lesions caused by burns, systemic lupus erythematosus, and herpes [39–41], protect cultured human neurons under oxidative stress [42], reduce ischemia-reperfusion injury [22, 43, 44], inhibit angiotensin II (AngII) intraneuronal signaling [19], prolong viability of *β*-cells [45], be effective in the treatment of rat adjuvant arthritis [46], and so forth. Most relevant to this study, antioxidants, including SOD, are also thought to be beneficial in the treatment of eye diseases. The eye is rather isolated organ, and the pathological processes within it are preferably treated not* via* systemic but by local drug intake. SOD1 was found in the corneas of mammals [47], suggesting that the enzyme plays an important role in maintaining homeostasis of the ocular surface. The use of topical, subconjunctival, parabulbar, or intraocular injections of SOD1 could, therefore, provide a supplement for intrinsic antioxidants in eye tissues, which may be depleted during inflammation.

According to official statistics, inflammatory eye diseases are the most common eye pathologies which lead to partial disability and, sometimes, to the complete loss of vision. Among these diseases, the most severe one is uveitis, inflammation of uveal tract involving both outer and inner structures of the eye. Both noninfectious and infectious uveitis are accompanied by the enhancement of free radicals formation and by the increase of the content of the products of lipid oxidation in eye tissues. The major role in ROS formation in the eye belongs to polymorphonuclear leukocytes, which initiate as well as perpetuate the membrane oxidative processes at uveitis. Thus uveitis is believed to be strongly associated with overproduction of ROS in the eye tissues during inflammation, and antioxidants can play beneficial role in the treatment of this disease [48]. It was shown [9] that superoxide production by the leukocytes of Behçet patients (with uveal inflammation) was significantly higher in the attack phase than in the remission phase. Leukocyte superoxide generation was also enhanced in guinea pigs with S-antigen-induced experimental autoimmune uveitis. These observations indicate the perspectives of the use of antioxidants, SOD1 in particular, as potential drugs in the treatment of uveitis.

Previous studies have shown beneficial effects of SOD1 in the treatment of eye inflammation, including uveitis and eye burns. Thus, animals with phacoanaphylactic endophthalmitis (lens-induced uveitis) were treated with SOD1 [8]. This treatment resulted in strong reduction of choroid inflammation, retinal edema, and vasculitis. In another study aqueous humor cell quantity and infiltration of the inflammatory cells in the anterior retina were markedly reduced in SOD1-treated animals with both S-antigen induced and bovine serum albumin-induced passive Arthus type uveitis [9]. Positive effect of SOD1 was also demonstrated on the rabbit model of immunogenic uveitis [14]. Moreover, topical antioxidant therapy by SOD1 in acute corneal inflammation (induced by alkali burn) was shown to be efficient in reduction of corneal ulcers [11], while subconjunctival injections of SOD1 were reported to prevent tissue destruction after alkali burns of the eye and prevented corneal perforation [10].

However, the methods of the treatment of the diseases, which include inner structures of the eye, are relatively less effective due to the poor transport of proteins and other drugs into the eye. In recent years, there has been significant interest in the developing nanosized drug delivery systems to overcome the limitations of drug therapy. Such nanosystems can improve the therapeutic efficacy of the drugs by overcoming diffusion barrier, by increasing their stability in biological tissues and fluids and enhancing cellular/tissue uptake. These nanosystems are attractive for the treatment of various eye diseases, including both acute and chronic conditions [17, 49]. So far, SOD1 entrapped in liposomes was previously shown to be effective in the treatment of noninfectious corneal ulcers [50].

Recently, a cross-linked polyion complex of SOD1 with a cationic block copolymer PEG-pLL_50_, termed “SOD1 nanozyme,” was developed [21, 22]. This SOD1 nanoformat is characterized by high dispersion stability, small particle size, particle uniformity, decreased cellular toxicity, and efficient transport into cells. SOD1-nanozyme was shown to be able to effectively scavenge ROS and decrease ischemia/reperfusion-induced tissue injury and improve sensorimotor functions in a rat middle cerebral artery occlusion model [22]. In this study, the therapeutic efficacy of SOD1 nanozyme for the treatment of ophthalmic inflammatory diseases was demonstrated in a rabbit model of immunogenic uveitis.

Immunogenic uveitis is an animal model of acute ocular inflammation induced by the intraocular injection of serum from a foreign animal after presensitization. This type of uveitis usually includes inflammation in the anterior, intermediate, and posterior segments of the eye, thus representing panuveitis. We hypothesized that antioxidant agent SOD1 in the form of nanozyme can attenuate oxidative stress and produce a significant therapeutic effect. For the treatment, we have chosen the most simple and convenient drug formulation, aqueous solution of nanozyme as eye drops.

In this study, we mostly followed clinical and biochemical parameters in the anterior segment of the eye in uveitis, while further histological study allowed us to estimate uveitis manifestations in the posterior segment of the eye as well. Clinical manifestations of uveitis in the anterior segment of the eye could be divided into two groups. The first are manifestations in the outer part of the segment including eyelid edema, eyelid hyperemia, conjunctival edema, conjunctival hyperemia, corneal edema, and neovascularization of the cornea. The second are manifestations in the inner part of the segment including iris edema, iris hyperemia, fibrin clots and precipitates on the iris and on the lens, lens opacity, the existence of synechiae, and the existence of exudates in the anterior chamber of the eye.

The major result of this study is a clear demonstration that topical instillations of SOD1 nanozyme solution into the eye exhibit remarkable effect on the clinical manifestation of the disease, improve biochemical characteristics of the aqueous humor, and help to maintain the cells of various eye tissues in normal condition. In the eyes of rabbits not receiving any treatment, but receiving placebo instead, we observed acute panuveitis. Inflammation included entire uveal tract, which, in turn, caused inflammation in other eye tissues. Deformation of almost every eye tissue was observed, retina (defects of photoreception apparatus), ciliary body (distortion in the aqueous humor formation and in accommodation), iris (changes in the structure and immobilization of the pupil), lens (opacity, cataract), and cornea (decrease of the transparency). In the second group of rabbits receiving native SOD1 solution, the clinical manifestations of uveitis were less severe. However, edema, inflammatory infiltration, and endothelium desquamation were still observed. In the third group of rabbits receiving SOD1 nanozyme solution the therapeutic effect of antioxidant agent was much more pronounced. We observed statistically significant differences in those clinical manifestations of uveitis, such as corneal and iris edema, hyperemia of conjunctiva, lens opacity, and amount of fibrin clots between this group and native SOD1-treated group. The biochemical characteristics of the aqueous humor were also improved. Moreover, histological study demonstrated almost normal structure of eye tissues from this group. Remarkably, the proposed therapy appears to be beneficial for treatment of not only the surface but also inner areas of the eye.

The current study did not allow precise delineating of the mechanism by which the nanozyme formulation improves the therapeutic effect of SOD1. The effect of the topical application of common drugs is greatly impeded by the protective physiological barriers of the eye, which effectively decrease the concentration of the drug in the site of the action [15]. Previous works have shown that incorporation of SOD1 in nanozyme format increases the efficacy of nanozyme delivery in cells [19, 51]. Moreover, the stability of the enzyme taken up into the cells within the nanozyme format is greatly increased, presumably, due to stabilization of the enzyme molecule against metabolic degradation and/or lysosomal escape [22]. Studies have also shown that nanozymes can be taken up in macrophages, when they can reside for considerable periods of time [18, 20]. Nanozymes can be also transported with macrophages to distal disease sites, where they are released into the extracellular media as well as within other tissue cells and exerted the protective effect by scavenging the ROS [52, 53]. All these effects could in principle contribute to improved therapeutic effect of the SOD1 nanozyme during uveitis observed in this work.

Altogether the results obtained demonstrate high potential therapeutic efficacy of topical administration of SOD1 nanozyme for the treatment of inflammatory eye diseases. Most current therapies of uveitis are predominantly based on steroids and immunosuppressants [54, 55]. However, steroids have systemic side effects such as cataract, glaucoma, and secondary ocular hypertension [56, 57], while immunosuppressive drugs are teratogenic and contraindicated during pregnancy [55]. Intraocular or periocular injections can deliver relatively high doses of drug to the eye with fewer side effects [54, 55]; however, each such injection is in essence a minor surgical procedure that could be quite disruptive and inconvenient to a patient. Recently, several sustained-release drug delivery implants have been developed to treat noninfectious uveitis, but such implantation requires surgical operation and the cost of this invasive treatment is high to the patients and insurance companies [55].

Therefore a noninvasive topical SOD1 nanoformat that can be conveniently applied as eye drops by a patient if shown successful as a therapeutic modality could be a major breakthrough in treatment of uveitis and possibly other inflammatory conditions of the eye.

## 5. Conclusions

In summary our work demonstrates that the nanozyme formed by self-assembly of the SOD1 with PEG-pLL_50_ block copolymer and stabilized by cross-linking can be used as a carrier for sustained delivery of SOD1 into ocular tissues for the treatment of inflammation processes in the eye. Topical instillations of SOD1-nanozyme significantly decreased inflammation both in the outer and inner parts of the eye as determined using scores of the clinical manifestations of uveitis, multiple biochemical parameters, and histological analysis. These results may have broad clinical implications in the treatment of other disorders of the eye where oxidative stress contributes to pathology.

## Figures and Tables

**Table 1:** Biochemical parameters of aqueous humor on day 8 of uveitis in rabbits.

Biochemical parameter	Treatment			
	Control	Placebo	SOD1	Nanozyme
Total protein concentration, mg/mL	2.2 ± 0.3	19.3 ± 3.2	15.6 ± 2.3	10.5 ± 1.1^*∗∗*^
*α*_2_-Macroglobulin, arb. U/mL	0.6 ± 0.1	10.8 ± 1.0	8.7 ± 1.5	7.2 ± 1.6^*∗*^
Antioxidant activity, U/mL	14.4 ± 1.0	2.3 ± 0.9	3.3 ± 0.6	3.7 ± 0.2
SOD1 activity, trolox-equivalents/mL	280 ± 40	460 ± 100	370 ± 80	330 ± 50^*∗*^

^*∗*^Significant difference between nanozyme and placebo by the Mann-Whitney *U* test, *p* < 0.05; ^*∗∗*^Significant difference between nanozyme and placebo, and nanozyme and native SOD1 by the Mann-Whitney *U* test, *p* < 0.01.
